# Does Structural Complexity Determine the Morphology of Assemblages? An Experimental Test on Three Continents

**DOI:** 10.1371/journal.pone.0064005

**Published:** 2013-05-17

**Authors:** Heloise Gibb, Catherine L. Parr

**Affiliations:** 1 Department of Zoology, La Trobe University, Melbourne, Victoria, Australia; 2 Swedish University of Agricultural Sciences, Department of Wildlife, Fish and the Environment, Umeå, Sweden; 3 Environmental Change Institute, School of Geography and the Environment, University of Oxford, Oxford, United Kingdom; 4 Department of Earth, Ocean and Ecosystem Sciences, School of Environmental Sciences, University of Liverpool, Liverpool, United Kingdom; University of Western Ontario, Canada

## Abstract

Understanding how species will respond to global change depends on our ability to distinguish generalities from idiosyncrasies. For diverse, but poorly known taxa, such as insects, species traits may provide a short-cut to predicting species turnover. We tested whether ant traits respond consistently to habitat complexity across geographically independent ant assemblages, using an experimental approach and baits. We repeated our study in six paired simple and complex habitats on three continents with distinct ant faunas. We also compared traits amongst ants with different foraging strategies. We hypothesised that ants would be larger, broader, have longer legs and more dorsally positioned eyes in simpler habitats. In agreement with predictions, ants had longer femurs and dorsally positioned eyes in simple habitats. This pattern was most pronounced for ants that discovered resources. Body size and pronotum width responded as predicted for experimental treatments, but were inconsistent across continents. Monopolising ants were smaller, with shorter femurs than those that occupied or discovered resources. Consistent responses for several traits suggest that many, but not all, aspects of morphology respond predictably to habitat complexity, and that foraging strategy is linked with morphology. Some traits thus have the potential to be used to predict the direction of species turnover, changes in foraging strategy and, potentially, evolution in response to changes in habitat structure.

## Introduction

An improved understanding of how species will respond to global change depends on our ability to distinguish generalities from idiosyncrasies in species responses. This is particularly true for diverse taxa, such as arthropods, for which a detailed understanding of the ecology of the estimated 2–10 million species [1], [2], [3] is probably untenable within this century. Trait-based approaches to understanding and predicting species responses to their biotic and abiotic environment are increasingly central to our understanding of the structure of ecological communities and the functional consequences of community disassembly [4], [5]. Traits thus offer the potential to better predict the responses of a diversity of insects to changes in their environment across a broader range of species than do species-focussed approaches.

Whilst species possess a large range of traits, including attributes of behaviour, phenology, dispersal and reproductive strategies, morphological traits provide the greatest potential to be easily measured across a large sample of poorly known species. Previous studies have shown clear relationships between morphology and function in insects. For example, wing morphology allows the prediction of insect dispersal ability [6], [7], while body size responds predictably to climate [8], [9], [10]. Morphological traits allow us to use evolutionary convergences resulting from niche similarities to determine the functional similarity of ecosystems that do not necessarily share species [4]. A traits-based approach therefore provides potential for a greater understanding of large-scale patterns.

Changes in the structure of habitats may be a significant component of global change, driven in particular by changes in climate and agricultural intensification [11], [12], [13], [14]. Morphologies differ between habitats differing in complexity, for example, ant leg length decreases with habitat complexity [15], [16], [17], [18], [19] and larger species are advantaged by simpler habitats [16], [17]. This suggests that morphological characters of assemblages of species might be expected to change with modification of habitat complexity caused by global change. This could occur both through species turnover and, in the longer term, through species’ evolution. Although localised species responses to reductions in habitat complexity are well documented, no studies have attempted to determine generalities in responses globally using a morphological approach.

Ants are abundant and functionally important in ecosystems worldwide [20], [21], but their high diversity limits our capacity to understand the response of individual species to global change. Although functional groups have commonly been used to explore the responses of ant assemblages to environmental change (including disturbance) [22], [23], [24], recent studies have used a more systematic approach based on morphology [25]. While categorical functional groups may provide useful rapidly applied measures in a localised area, continuous measures of assemblage traits are likely to be more sensitive to a gradient of environmental change [4]. Here, we use experimental and mensurative approaches to test the generality of morphological trait responses to habitat complexity in sites on three continents. We targeted morphological traits related to body size and proportions, relative leg length and eye position, which have previously been shown to respond to habitat complexity at local scales [16], [17], [25], [26], [27]. We also considered whether morphology is related to foraging strategy. We hypothesised that, in simpler habitats, where movement and vision are not inhibited, species would be larger and broader, with relatively long legs, large heads and more dorsally positioned eyes than species in more complex habitats. We expected that ant species that were first to discover resources would have longer legs than species that eventually monopolised the same resources. We predicted that responses would be consistent among assemblages from different continents and using different experimental approaches.

## Materials and Methods

### Ethics Statement

All necessary permits were obtained for the described field studies. Permits were obtained from the National Parks and Wildlife Service of NSW, Australia, Västerbotten Länstyrelsen, Sweden, and South African Parks, South Africa.

### Study Areas

The study was conducted in sites in South Africa, Australia and Sweden (Fig. S1). Each of these regions supports a distinct ant fauna due to disparate geological histories, although Australian and South African faunas are more similar to one another as a result of their shared Gondwanan past. In using distinct ant faunas, we aimed to determine if there were generalities in the responses of ant traits to habitat complexity. We did *not* aim to determine generalities about particular parts of the world from this study. Within each region, six study sites with paired complex and simple habitats in close proximity (within 2 km of one another) were selected. Using methods detailed in Gibb and Parr [18], we quantified microhabitat structure for the simple and complex habitats in each region (Table S1).

### Habitat Complexity and Competition Experiment

The hypothesis that ant morphological traits respond predictably to habitat complexity was tested using an experimental set-up similar to that of Sarty et al. [17]. Experiments were conducted in Sweden in July 2007, in South Africa in December 2007 and in Australia in February 2008. All sampling was conducted during the summer in each region. We were interested in determining if there were general patterns in responses to habitat complexity shared among regions. The role of microhabitat complexity was tested using two experimental treatments: “fine” and “coarse” and two control treatments: “control” and “bait card” i.e. a total of four treatment types. Three experimental blocks consisting of one replicate of each of these treatments were placed on each simple and complex habitat each morning of the experiment (9∶00–13∶00). Sampling was continued in the afternoon (13∶00–18∶00) in three new, independent experimental blocks at the same site (Fig. S1). In total, six replicates of each treatment were examined in each habitat at each of the six sites in simple and complex natural habitats, making a total of 36 replicates for each of the four treatments and two habitats in each of the three regions i.e. n = 864.

Experimental treatment arenas were constructed using round transparent plastic containers (diameter: 18 cm; height: 9 cm), containing a central observation chamber [17], [18], [28] (Fig. S2). “Control” treatments were containers with no added structure, while “bait card” treatments consisted of a microscope slide with a bait card attached. Entrance holes to chambers were 1 cm in diameter, which allowed entry to all ant species, except the bull ant *Myrmecia gulosa*, which was relatively rare in the study sites in Australia. Containers for the “fine” and “coarse” treatments were filled with natural materials to create structure. Materials selected included the most common herb species, branches of shrub-layer plant species, leaf litter, cones and rocks. Cotton wool was packed on top of these materials to prevent ants walking over the top of the experimental habitat. Gap size was measured by placing a regular grid of 18 points over the experimental microhabitat (depth 7 cm), held over a light source. Gaps closest to each of the 18 points were traced to a transparency and measured to the nearest 0.5 mm. Amongst regions, gap sizes varied between (mean ± SE) 1.0±0.1 mm and 2.8±0.1 mm for the fine treatment and 6.1±0.6 mm and 14.1±2.1 mm for the coarse treatment [14]. Between replicate runs, containers used as controls and coarse and fine treatments were haphazardly reallocated to treatments and containers were repacked. Different treatments were performed simultaneously so there was limited opportunity for temperature to confound the effects of treatments.

Ants were able to access the experimental arena through 24 holes, each 1 cm in diameter, at the base of the container. Baits of honey (contained in a vial lid, 2 cm in diameter) and fish-based cat food (pieces approximately 1.5 cm in diameter) were placed on a laminated bait card in the central observation chamber and replenished *ad libitum*. A petri-dish covered the opening to the observation chamber.

All experimental arenas and bait cards were observed for a total of three hours after placement in the field. Replicates were observed until they had been discovered. The time taken until discovery of each bait was recorded and the first ant to discover it was collected. The temperature, the identity of the ants, their abundance, and the bait on which they were feeding was recorded each hour. After three hours, specimens of the ants present on the baits were collected. A forager was classified as “discoverer” if it was first to find a resource, “occupier” if it was present at a resource at 3 hrs, and “monopoliser” if, at 3 hrs, it was the only species present at a bait and five or more workers were present. These categories are referred to as “foraging strategies”, although a single species may have been classified in all three categories. One and two hour observations were not analysed.

### Morphometric Measurements

Six specimens of each species collected were selected for measurement, except when fewer specimens of the species were observed. In dimorphic species, only minors were used as majors were relatively rare. For each ant, standard linear measurements were taken using an ocular micrometer mounted on a dissecting microscope accurate to 0.01 mm. These measurements were head width across the top of the eyes and between the eyes, head length from the posterior edge of the clypeus to the posterior cephalic margin, hind femur length, Weber’s length ([29]; a measure of body size based on the alitrunk) and pronotum width. To determine the position of ant eyes relative to the side of the head, referred to here as ‘eye position’, we subtracted the distance between the eyes from the head width across the top of the eyes. This is distinct from Silva and Brandão’s [25] ‘eye position’, which was measured as distance from the mandibles.

### Data Analysis

Because we directly targeted traits for which we predicted specific relationships with habitat structure, and which potentially act as indicators of assemblage change, we used a univariate approach to analysing our data. Residuals of the regression of each trait on Weber’s length were used for all analyses because regression analysis showed that all traits were strongly and significantly (*p*<0.0001) positively related to Weber’s length: eye position r^2^ = 0.65; all others r^2^>0.8. Principal components axes obtained from the five traits did not reveal interpretable correlations between traits other than with respect to body size, so residuals were considered more meaningful. The residualised traits were not significantly correlated amongst species (r^2^<0.02), except for head length, which was correlated with pronotum width (r^2^ = 0.30, *p*<0.0001). Head length was discarded from further analyses and all traits were considered independent. Means across the six replicates of each region, site, habitat and treatment combination were used. Means were weighted by occurrence in the six replicates, but not by abundance.

Linear mixed effects models (LMM) using a restricted maximum likelihood estimation (REML) approach on JMP [30] and post-hoc Tukey’s tests were used to test our main hypotheses. A full model with the predictors treatment (fixed), habitat (fixed) and region-site (random; n = 3 regions×6 sites = 18) (e.g., Weber’s length ∼ habitat+treatment+habitat*treatment, random = region-site) was used to test effects on the five trait variables for ants that discovered resources, that occupied resources at 3 hrs and that monopolised resources at 3 hrs. Treating region as a random factor in the LMM meant that we could not obtain any insights into how the effects of habitat differed between the specific regions in which we sampled. We therefore also tested the effects of a full model that included region (fixed), habitat (fixed) and site (region) (random) (e.g., Weber’s length ∼ habitat+region+habitat*region, random = site(region)) using the mean across the four treatments for the four trait variables. ANOVA was used to test the effect of region, foraging strategy (i.e., ants that were first to discover or occupied or monopolised at three hours) and their interaction on ant traits.

## Results

At three hours, baits were occupied by 28 different species/morphospecies in S Africa (22 in complex; 13 in simple habitats), 24 in SE Australia (19 in complex; 16 in simple) and 8 in Sweden (6 in complex; 8 in simple) (Table S2). In S Africa, baits were commonly monopolised by species of *Pheidole* and *Monomorium*; in SE Australia by species of *Crematogaster*, *Iridomyrmex*, *Monomorium* and *Ochetellus*; and in Sweden by species of *Formica* and *Myrmica*. Here, we focus on trait-based, rather than species-specific, responses.

### Trait Responses to Experimental Habitat Complexity

**Figure 1:**
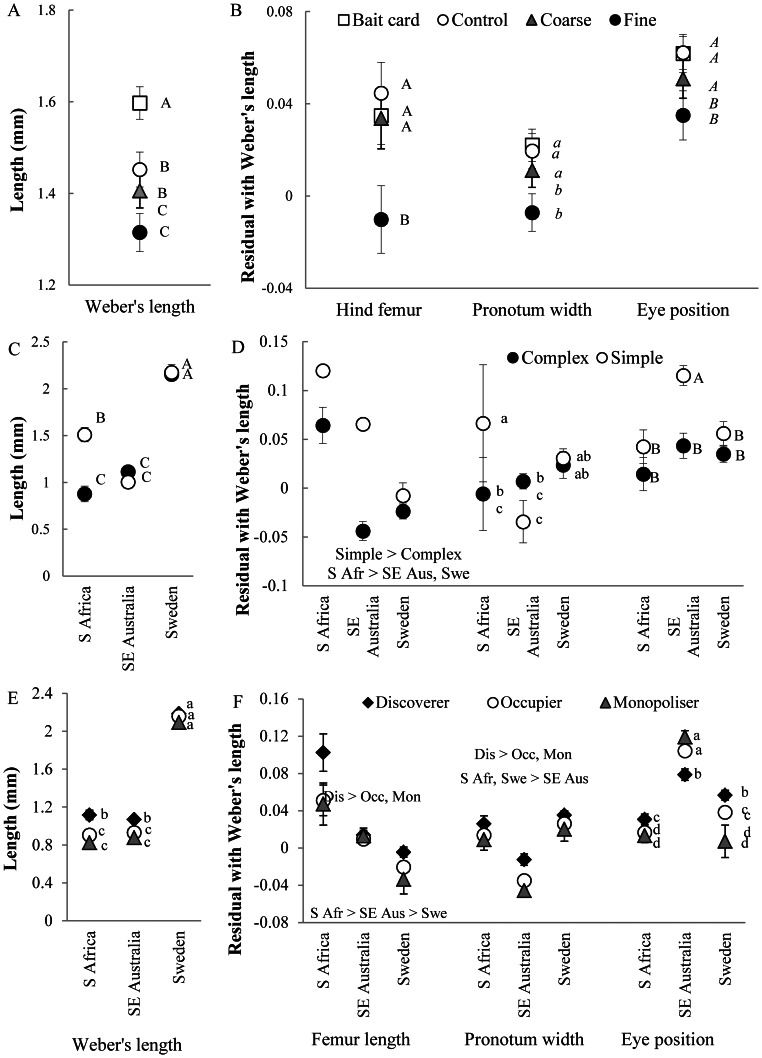
Mean ± SE measures for morphological traits of ants that were first to discover baits (‘discoverer’) in *experimental treatments*. a) Weber’s length; and b) residuals of hind femur, pronotum width and eye position with Weber’s length; ants that were first to discover baits in *natural habitats among regions*: c) Weber’s length; and d) residuals of hind femur, pronotum width and eye position with Weber’s length; and comparing species that were first to discover a resource, those that occupied it at 3 hours and those that monopolised it for: e) Weber’s length; and f) residuals of hind femur, pronotum width and eye position with Weber’s length. Different letters indicate significantly different means. Results for ants occupying and monopolising resources at three hours were similar to those that were first to discover them (Figs. S4,5).

The effects of experimental habitat complexities for discoverers, occupiers and monopolisers were largely similar to those of natural habitat complexity (Table 1, Fig. 1). Treatment effects were consistent with expectations, with the magnitude of trait means changing along a gradient from bait cards to controls to coarse to fine treatments, although not all differences were significant (Table 1, Figs. 1 a,b, Fig. S3). Patterns were particularly clear for Weber’s length for discoverers and occupiers. Ants that successfully accessed food resources (i.e., they discovered, occupied or monopolised resources) in fine treatments had a relatively short head and femur and a relatively narrow pronotum. Eyes of these ants were positioned more laterally. The only significant interaction between natural and experimental habitat complexities was for monopolisers. Post-hoc Tukey tests showed that Weber’s length was shorter, i.e., monopolisers were smaller, in fine treatments, but only in simple habitats (Table 1, Fig. S3).

**Table 1:** F-values from REML ANOVA testing the effect of: a) habitat, treatment and their interaction (df = 1,3,3); and b) habitat, region and their interaction (bait cards only; df = 1,2,2) on morphological features of ants.

source	Weber’s L	femur L	PronotumW	eye position	
**a) ** ***treatment x habitat***					
**discovering ant**					
habitat	15.30***	17.20***	0.30	50.84***	
treatment	9.60***	2.51†	2.46†	3.05*	
treatment*habitat	1.22	0.73	0.35	0.27	
**occupying ants**					
habitat	46.73***	13.79***	0.54	72.11***	
treatment	4.71**	0.99	2.87*	3.03*	
habitat*treatment	1.64	1.56	0.52	1.38	
**monopolising ants**					
habitat	23.40***	6.02*	0.09	43.70***	
treatment	3.06*	0.33	0.97	1.27	
habitat*treatment	3.13*	2.55†	0.97	1.02	
**b) ** ***habitat*** * x * ***region***					
**discovering ant**					
habitat	20.35***	7.38**	1.00	25.65***	
region	170.98***	6.32*	5.34*	8.63**	
habitat*region	31.03***	1.46	11.23***	3.98*	
**occupying ants**					
habitat	165.06***	2.21	7.97**	15.10***	
region	38.10***	5.84*	1.47	27.77***	
habitat*region	24.52***	0.92	2.20	3.86*	
**monopolising ants**					
habitat	38.02***	1.05	0.01	27.81***	
region	165.26***	0.23	12.33***	15.11***	
habitat*region	24.44***	21.01***	1.60	3.85*	

Analyses were performed for the first ant to discover the bait, the “discovering ant”, across all ants present at three hours “occupying ants” and for those ants that monopolised the bait, with five or more workers present, “monopolising ants”. All measures except Weber’s length are based on residuals with Weber’s length. L = length; W = width. Values shown are F values. Results for post-hoc t-tests are shown in Fig. 1. Significance levels:

† P<0.07,

* P<0.05,

** P<0.01,

*** P<0.001.

### Trait Responses to Natural Habitat Complexity Among Regions

Interactions between region and natural habitat complexity were significant for many traits for discoverers, occupiers and monopolisers. Responses of traits to natural habitat complexity, although consistent with predictions overall, thus differed among regions (Table 1). Weber’s length was greater in simple habitats only for ants in the S African sites (Table 1, Fig. 1c). Eyes were consistently more laterally positioned in complex than simple habitats, but not always significantly so. Hind femurs were relatively longer in simple than complex habitats for ants that discovered and occupied resources, but for monopolisers, the opposite pattern was observed in the S African sites (Table 1, Fig. 1d, Fig. S4). Ants in the Swedish sites were generally larger than in other regions, while ants in the Australian site had a relatively narrow pronotum and more dorsally positioned eyes (Table 1, Fig. 1d).

### Trait Differences Amongst Discoverers, Occupiers and Monopolisers

All traits differed between ants that discovered resources and those that occupied or monopolised them, with occupiers sometimes intermediate. Ants that discovered resources were consistently larger than those that occupied them at three hours or monopolised them (Table 2, Fig. 1e), although this was not significant for the Swedish sites, where there were relatively few species. Ants that discovered resources also had relatively longer femurs and narrower pronota than those that occupied or monopolised them (Fig. 1f). While we found a similar pattern for eye position in S Africa and Sweden, ants that discovered resources in SE Australia had more laterally positioned eyes than occupiers or monopolisers, contrary to predictions.

**Table 2:** F-values from ANOVA testing the effect of region, foraging strategy and their interaction (df = 2,2,4) on morphological features of ants.

source	Weber’s L	femur L	pronotum W	eye position
foraging strategy	21.67***	4.46*	7.07***	0.88
region	769.88***	27.81***	60.38***	135.80***
region*foraging strategy	2.72*	1.72	0.63	9.85***

All measures except Weber’s length are based on residuals with Weber’s length. L = length; W = width. Values shown are F values. Results for post-hoc t-tests are shown in Fig. 1. Significance levels:

* P<0.05,

** P<0.01,

*** P<0.001.

## Discussion

This study provides experimental and observational evidence of both generalities and idiosyncracies across continents in the responses of a range of morphological traits of ant assemblages (or the subset attracted to baits) to habitat complexity. We also observed distinct differences between ants that discovered resources and those that eventually monopolised them. These broad patterns, measured across phylogenetically disparate assemblages (see Table S2), suggest that some elements of community turnover in response to changes in habitat complexity across the globe may be predictable. This finding has implications for our ability to predict the response of assemblages to a variety of anthropogenic disturbances, which can result in the loss of biomass and changes in habitat complexity [13], [14].

Clear morphological differences between species that discovered and monopolised resources were apparent. Species that discovered resources were consistently larger than those that occupied them at three hours or eventually monopolised. This pattern was not significant in Sweden, possibly because species richness and turnover was low. Larger species often move faster than smaller species [31], so may discover resources first. Discoverers also had relatively longer legs, supporting a role for rapid exploration of the environment. The only trait response that behaved inconsistently between regions was eye position, which was more dorsal for discoverers in all regions, but SE Australia, where it was lowest. This pattern reflects the morphology of fast-moving species, which have eyes positioned to allow them a broader view of their environment. In SE Australia, eyes tended to be more dorsally positioned across the entire assemblage, driven mainly by *Ochetellus* and *Iridomyrmex*. These genera all recruit aggressively to resources, so monopolised resources at three hours, when more opportunistic genera, such as *Rhytidoponera*, were less successful. In other continents, smaller species, particularly myrmicines, which have shorter legs and more laterally positioned eyes, and are commonly superior recruiters and may possess chemical defences [32], were more successful monopolisers.

Although discovering, occupying and monopolising ants differed consistently in a number of traits, responses of traits amongst those groups to natural and experimental habitat complexity were largely consistent. In simple habitats, ants had relatively longer legs and eyes in more dorsal positions. Treatment and natural habitat complexities thus had very similar effects for these traits, suggesting that the treatments accurately mimicked components of the natural habitat. This provides strong support for a filtering effect of habitat complexity on the composition of species through their morphological traits. However, other traits responded as predicted in either treatment or natural habitats but not both: body size was greater in simpler habitats, but this was only true for natural habitats in S Africa, while pronotum width behaved consistently in treatments, but not natural habitats.

This is the first study to report a globally consistent and convincing relationship between the sensory morphology of entire assemblages and habitat complexity. Eye morphology has previously been shown to relate to time of activity (i.e. nocturnal, crepuscular or diurnal, [33], [34], [35], [36]), while eye position (measured as distance to the mandibles) may be determined by the degree of predatory behaviour [25], [37]. We show here that habitat complexity also determines the success of species, based on the position of their eyes. Species with lateral eyes, such as *Pheidole*, are more likely to be travelling through more complex habitats, for example moving under leaf litter, where it is not possible to see far ahead. Those with dorsal eyes are faster-moving, such as species of *Iridomyrmex*, living predominantly in open habitats. Turnover in species, favouring those with more dorsally positioned eyes, is thus a likely response to simplification of habitats.

Femurs were proportionally smaller in more complex habitats and the fine treatment. This is in addition to (and not confounded by) the response described by the size-grain hypothesis, which suggests that smaller organisms perceive the earth’s surface as more rugose than larger organisms [26], [38], [39], [40]. Our study suggests that variation in the scaling of body size and leg length is also driven by the complexity of the habitat in which a species dwells. Thus, although body size imposes some limitations, small species can dwell in simple habitats if their legs are proportionally longer, while large species with relatively shorter legs may dwell in complex habitats. Relative femur lengths were greater in Africa, compared with other sites, which may result from lower overall rugosity in the microhabitats examined.

The relationship between habitat complexity and Weber’s length reflects the association between body size and habitat complexity observed in a range of taxa and systems [17], [41], [42], although not all [15]. The mechanism through which this operates is likely to be restriction of the movement of larger species allowing smaller species to thrive [43]. This is reflected in slower rates of discovery in complex habitats, where movement of larger ants is impeded, such that only smaller ants with relatively shorter legs are physically able to access resources [18]. While responses to treatments were consistent with predictions, it is unclear why we detected this pattern in natural habitats only for the S African sites. The high similarity in species composition may have obscured differences between simple and complex sites in Sweden, while a strong effect of habitat complexity on femur size in Australia may compensate for the similarity in body size.

For pronotum width, effects were inconsistent for natural and treatment complexities and between regions. It is possible that the mixed response to the different tests reflects a weaker relationship to habitat complexity that is context-dependent. Phylogeny and limitations of the species pool are likely to be drivers of the idiosyncratic patterns. In particular, the variable effects of natural habitats on Weber’s length and pronotum width may be a result of differences in the basic morphology of the taxa present at this limited set of sites. For example, dolichoderine ants, particularly species of *Iridomyrmex*, dominate open habitats in Australia [14], [22], [24], [44] and have relatively narrow pronota and long legs that may compensate for relatively small body size. In contrast, species occupying open habitats in Sweden and Africa included formicine genera such as *Formica* and *Camponotus* with relatively broad pronota and medium to large body size. Habitat complexity may thus favour the success of different traits, depending on phylogenetic context and trait associations. These idiosyncrasies in the response of traits to natural habitat complexity between sites suggest that a broader geographic coverage, both within and between regions, is required to fully understand how species perceive their habitats.

A further issue faced by studies that use multi-scalar manipulative approaches to understanding community structure is that responses to natural and treatment habitats may differ due to differences in the filtering effects of habitat complexity at different scales and the distinct problems inherent in experimental and mensurative tests [45], [46]. While treatments acted as a filter on the contemporary fauna, natural habitats have to some extent already filtered their fauna through evolutionary processes that determine the regional species pool. As is the case in most mensurative studies, habitats differing in complexity may also have differed in a range of other factors, e.g., resource availability and temperature. On the other hand, experimental artefacts may have influenced outcomes in treatments, e.g., if ants were inhibited from entering the experimental chambers. Thus, when both methods achieved similar outcomes, the results can be considered convincing, while less consistent results require further investigation before stronger inferences can be drawn.

### Conclusions

The diverse assemblages sampled responded predictably to manipulated habitat complexities in a way that was often consistent with responses to natural habitats and with our predictions. Idiosyncrasies between regions in the response of traits to natural habitats suggest that some of the morphological traits measured may not respond strongly to habitat complexity and that further work is required to better understand the other factors driving morphology at both regional and global scales. Turnover of femur length and eye position in response to habitat complexity was consistent with predictions across disparate species assemblages. This suggests species composition within a regional or local assemblage changes in response to habitat complexity, with species success dependent on traits. The value of these traits depended upon the foraging strategies of species. For example, short femur length might limit discovery of a resource in an open habitat, but the ability to recruit to that resource might allow eventual monopolisation by a short-legged species. Over a longer timeframe, changes in habitat complexity may also drive changes in species traits through the evolution of individual species to better match their habitats. A range of global change drivers alter habitat complexity, including anthropogenic habitat disturbance and modification, climate change and invasive species. Turnover in species assemblages, and even extinctions of some species, are thus likely to result from global change. While we lack the resources to predict outcomes for all species, it is critical that we can predict which types of species will be negatively affected. Here, we have shown strong relationships between habitat complexity and particular morphological traits. The use of such traits provides us with a tool with which we can determine this risk without a detailed understanding of the biology of all 2–10 million insect species.

## Supporting Information

Figure S1 Design of the experiment, showing anticlockwise from top right: a) localities within regions (NR nature reserve; NP national park); b) paired layout of within-locality sites of high and low complexity; c) layout of treatments within sites; d) set-up for replicate sets of experimental bait chambers.
(TIF)

Figure S2 Example of experimental habitat chambers used in the study: those shown are for the Australian low complexity macrohabitat. We used a) a bait card control; b) control chambers contained no fill; c) low complexity or “coarse” habitat chambers contained sticks and banksia cones; and d) high complexity or “fine” habitat chambers contained densely packed vegetation. Habitat materials were substituted for local materials in each study location. Ants entered the habitat complexity chamber through holes in the outer container and the central observation chamber, which held the baits, through holes in the inner cup.
(TIF)

Figure S3 Mean ± SE measures for morphological traits of ants that occupied baits after three hours in experimental treatments: a) Weber’s length; b) residuals of hind femur, pronotum width and eye position with Weber’s length (“residual measures”); and ants that monopolised baits after three hours: c) Weber’s length showing interactions between natural habitats and experimental treatments; and: d) residual measures in experimental treatments. Different letters indicate significantly different means.
(TIF)

Figure S4 Mean ± SE measures for morphological traits of ants that occupied baits at three hours in natural habitats among regions: a) Weber’s length; and b) residuals of hind femur, pronotum width and eye position with Weber’s length (“residual measures”); and of ants that monopolised baits at three hours in natural habitats among regions: c) Weber’s length; and d) residual measures. Different letters indicate significantly different means when interactions were significant. Results of post-hoc tests are described in words where there was no interaction.
(TIF)

Table S1 Description of the simple and complex study sites in each region, with dominant vegetation types and mean ± SE litter depth and percentage bare ground (modified from Gibb & Parr, 2010).
(DOC)

Table S2 Occurrence of species at baits after 3 hours in complex and simple natural habitats in each of the regions. Abbreviations for morphological traits are: WL: Weber’s length; HL: head length; FL: femur length; PW: pronotum width; HW1: head with between the eyes; HW2: head width behind the eyes; EP: eye position (EP = HW2-HW1).
(DOC)
