# Supplementary figures and images for: Does Structural Complexity Determine the Morphology of Assemblages? An Experimental Test on Three Continents
Source: PLoS One. 2013 May 17;8(5):e64005. doi: 10.1371/journal.pone.0064005 (PMC3656910; doi:10.1371/journal.pone.0064005)

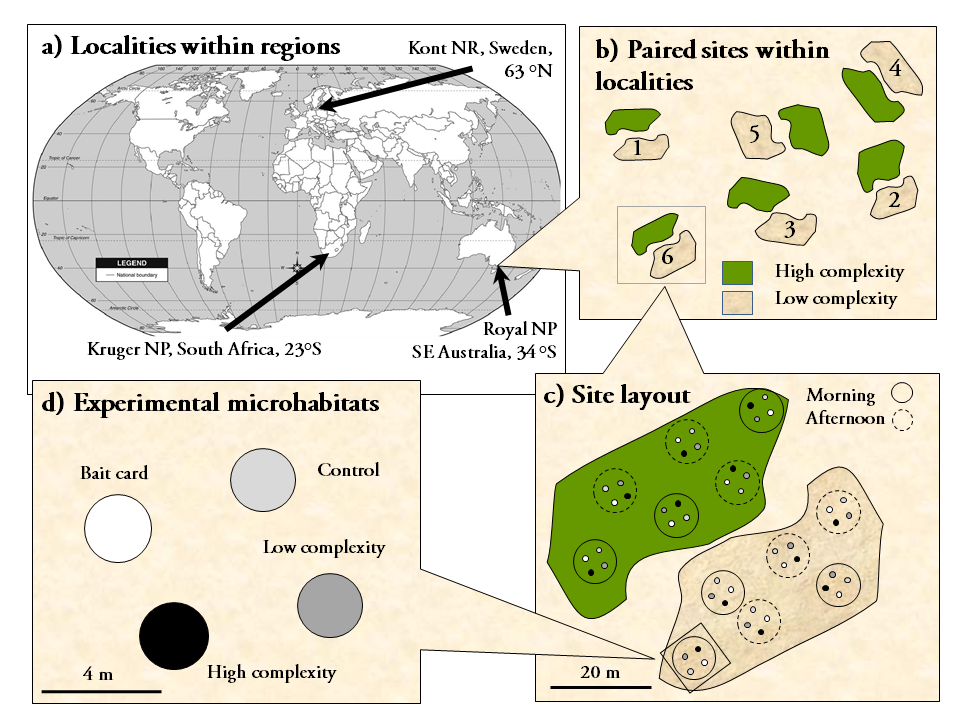

Supplement: Figure S1 — Design of the experiment, showing anticlockwise from top right: a) localities within regions (NR nature reserve; NP national park); b) paired layout of within-locality sites of high and low complexity; c) layout of treatments within sites; d) set-up for replicate sets of experimental bait chambers. (TIF) [file pone.0064005.s001.tif]

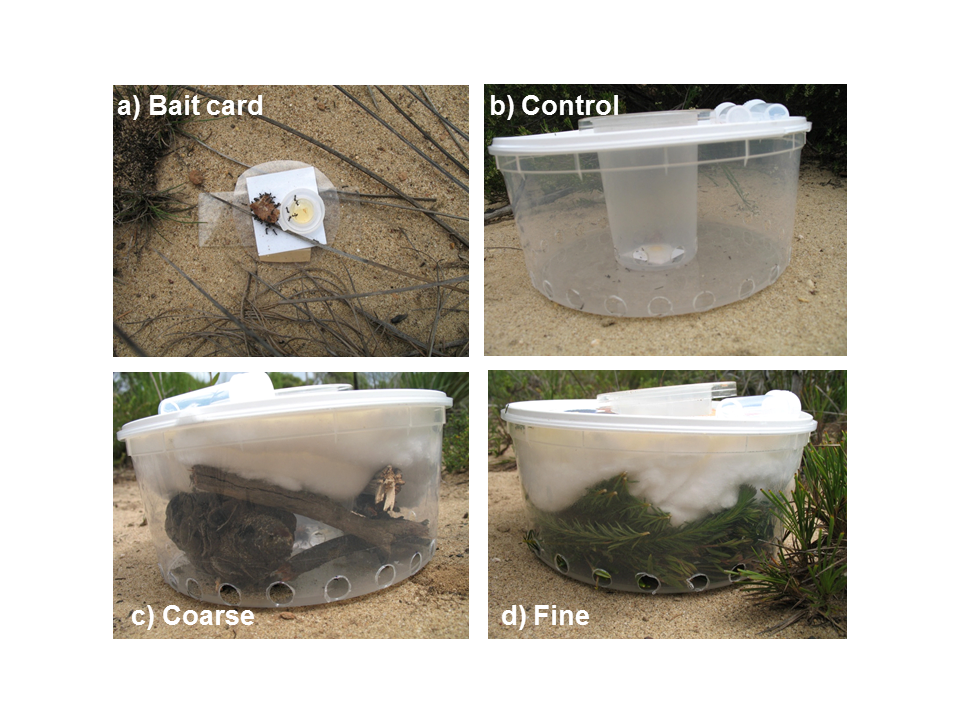

Supplement: Figure S2 — Example of experimental habitat chambers used in the study: those shown are for the Australian low complexity macrohabitat. We used a) a bait card control; b) control chambers contained no fill; c) low complexity or “coarse” habitat chambers contained sticks and banksia cones; and d) high complexity or “fine” habitat chambers contained densely packed vegetation. Habitat materials were substituted for local materials in each study location. Ants entered the habitat complexity chamber through holes in the outer container and the central observation chamber, which held the baits, through holes in the inner cup. (TIF) [file pone.0064005.s002.tif]
