# Supplementary material for: Does Structural Complexity Determine the Morphology of Assemblages? An Experimental Test on Three Continents
Source: PLoS One. 2013 May 17;8(5):e64005. doi: 10.1371/journal.pone.0064005 (PMC3656910; doi:10.1371/journal.pone.0064005)
Supplement: Table S1 — Description of the simple and complex study sites in each region, with dominant vegetation types and mean ± SE litter depth and percentage bare ground (modified from Gibb & Parr, 2010). (DOC) [file pone.0064005.s005.doc]

**Table S1:** Description of the simple and complex study sites in each region, with dominant vegetation types and mean ± SE litter depth and percentage bare ground (modified from Gibb & Parr, 2010).

| **Location** | **Complexity** | **Vegetation description** | **Litter depth (cm)** | **% Bare ground** |
| --- | --- | --- | --- | --- |
| Sweden:  Kont Nature Reserve  63.66 ºN, 20.22 ºE | Simple | Flat outcrops of gneiss sparsely covered in lichens (*Cladonia* spp.), surrounded by *Pinus sylvestris* forest | 0.03 ± 0.01 | 0.31 ± 0.03 |
|  | Complex | Lingonberry (*Vaccinium vitis-idaea***)**, bilberry (*V. myrtillus*) and moss (e.g. *Polytrichum commune*) understorey; *Pinus sylvestris* overstorey | 0.20 ± 0.08 | 0.00 ± 0.00 |
| South Africa:  Kruger National Park  25.01 ºS, 31.31 ºE | Simple | Sodic site, sparse cover of *Sporobolus* and *Cynodon* grasses and forbs. Large areas of bare ground. | 0.03 ± 0.01 | 25.30 ± 1.05 |
|  | Complex | Riverine habitat, dense ground layer of the forb *Acalypha indica*, *Panicum* grass and leaf litter | 0.60 ± 0.05 | 4.50 ± 1.48 |
| Australia:  Royal National Park  34.08 ºS, 151.10 ºE | Simple | Open heathland. Bare ground interspersed between shrubs (*Banksia* sp, *Grevillea sphacelata*, *Leptospermum arachnoides*) and grasses. | 0.10 ± 0.02 | 36.20 ± 1.30 |
|  | Complex | Copse woodland with a variety of trees (*Eucalyptus haemastoma racemosa*, *Corymbia gummifera*, *Banksia marginata*), shrubs (*Xanthorrhoea resinifera*) and a litter layer. | 1.90 ± 0.18 | 0.70 ± 0.49 |
