# Supplementary material for: Does Structural Complexity Determine the Morphology of Assemblages? An Experimental Test on Three Continents
Source: PLoS One. 2013 May 17;8(5):e64005. doi: 10.1371/journal.pone.0064005 (PMC3656910; doi:10.1371/journal.pone.0064005)
Supplement: Table S2 — Occurrence of species at baits after 3 hours in complex and simple natural habitats in each of the regions. Abbreviations for morphological traits are: WL: Weber’s length; HL: head length; FL: femur length; PW: pronotum width; HW1: head with between the eyes; HW2: head width behind the eyes; EP: eye position (EP = HW2-HW1). (DOC) [file pone.0064005.s006.doc]

**Table S2:** Occurrence of species at baits after 3 hours in complex and simple natural habitats in each of the regions. Abbreviations for morphological traits are: WL: Weber’s length; HL: head length; FL: femur length; PW: pronotum width; HW1: head with between the eyes; HW2: head width behind the eyes; EP: eye position (EP=HW2-HW1).

|  | **Habitat** | | **Morphological trait** | | | | | | |
| --- | --- | --- | --- | --- | --- | --- | --- | --- | --- |
| **Species** | **Complex** | **Simple** | **WL** | **HL** | **FL** | **PW** | **HW1** | **HW2** | **EP** |
| **S Africa** |  |  |  |  |  |  |  |  |  |
| *Camponotus cinctellus* | 1 | 18 | 2.21 | 1.57 | 2.29 | 0.95 | 0.86 | 1.20 | 0.34 |
| *Camponotus sp. A* | 5 |  | 2.33 | 1.52 | 1.89 | 1.47 | 0.98 | 1.57 | 0.58 |
| *Cardiocondyla sp. A* | 2 |  | 0.40 | 0.37 | 0.36 | 0.22 | 0.25 | 0.33 | 0.07 |
| *Lepisiota sp. A* | 18 | 1 | 0.70 | 0.37 | 0.67 | 0.37 | 0.33 | 0.42 | 0.09 |
| *Lepisiota sp. B* | 15 |  | 0.76 | 0.51 | 0.74 | 0.37 | 0.33 | 0.42 | 0.09 |
| *Meranoplus sp. A* | 12 |  | 0.41 | 0.81 | 0.60 | 0.83 | 0.75 | 0.76 | 0.01 |
| *Monomorium sp. E* | 1 |  | 0.70 | 0.68 | 0.62 | 0.60 |  | 0.66 |  |
| *Monomorium sp. A* |  | 86 | 0.78 | 0.68 | 0.49 | 0.38 | 0.49 | 0.59 | 0.11 |
| *Monomorium sp. B* | 2 | 5 | 0.35 | 0.34 | 0.16 | 0.18 | 0.21 | 0.26 | 0.05 |
| *Monomorium sp. D* | 2 |  | 0.46 | 0.44 | 0.34 | 0.22 | 0.30 | 0.35 | 0.05 |
| *Myrmicaria natalensis* | 17 | 48 | 2.17 | 1.60 | 2.71 | 1.30 | 1.38 | 1.85 | 0.47 |
| *Odontomachus sp. A* | 3 |  | 2.83 | 2.39 | 2.55 | 1.08 | 1.52 | 1.97 | 0.45 |
| *Pachycondyla sp. A* |  | 14 | 1.59 | 1.21 | 1.27 | 0.82 | 1.04 | 1.30 | 0.26 |
| *Pachycondyla sp. B* |  | 3 | 1.95 | 1.33 | 1.25 | 0.63 | 0.67 | 1.16 | 0.48 |
| *Pheidole megacephala* | 96 | 7 | 0.74 | 0.64 | 0.55 | 0.33 | 0.51 | 0.57 | 0.07 |
| *Pheidole sp. A* | 1 |  | 1.21 | 0.84 | 1.35 | 0.48 | 0.65 | 0.73 | 0.08 |
| *Pheidole sp. B* | 107 | 2 | 0.35 | 0.48 | 0.41 | 0.28 | 0.37 | 0.43 | 0.07 |
| *Pheidole sp. C* | 30 | 5 | 0.74 | 0.60 | 0.77 | 0.35 | 0.49 | 0.53 | 0.04 |
| *Pheidole sp. E* | 1 | 3 | 1.21 | 0.96 | 1.45 | 0.59 | 0.82 | 0.93 | 0.11 |
| *Pheidole sp. F* | 13 |  | 0.77 | 0.61 | 0.68 | 0.34 | 0.48 | 0.55 | 0.07 |
| *Pheidole sp. G* |  | 5 | 0.89 | 0.63 | 0.90 | 0.45 | 0.60 | 0.69 | 0.08 |
| *Plagiolepis sp. A* | 4 |  | 0.33 | 0.29 | 0.28 | 0.21 | 0.18 | 0.26 | 0.09 |
| *Plagiolepis sp. B* | 8 |  | 0.37 | 0.36 | 0.32 | 0.20 | 0.24 | 0.31 | 0.07 |
| *Polyrhachis sp. A* | 8 |  | 2.85 | 2.33 | 3.04 | 1.43 | 1.23 | 1.82 | 0.59 |
| *Technomyrmex sp. A* | 5 |  | 0.52 | 0.56 | 0.57 | 0.35 | 0.34 | 0.51 | 0.17 |
| *Tetramorium sp. B* | 2 |  | 0.60 | 0.58 | 0.47 | 0.36 | 0.43 | 0.50 | 0.07 |
| *Tetramorium sp. C* |  | 3 | 0.96 | 0.79 | 0.96 | 0.48 | 0.61 | 0.71 | 0.10 |
| *Tetramorium sp. D* | 2 |  | 0.60 | 0.60 | 0.51 | 0.39 | 0.53 | 0.65 | 0.12 |
| **SE Australia** |  |  |  |  |  |  |  |  |  |
| *Crematogaster sp. A* | 69 | 1 | 1.01 | 0.86 | 0.87 | 0.51 | 0.80 | 0.86 | 0.06 |
| *Dolichoderus sp. A* | 1 |  | 1.45 | 0.94 | 1.17 | 0.54 |  | 0.74 |  |
| *Dolichoderus sp. B* | 1 |  | 2.17 | 1.51 | 1.79 | 1.09 | 1.02 | 1.33 | 0.30 |
| *Iridomyrmex sp. A* |  | 15 | 1.44 | 1.04 | 1.72 | 0.58 | 0.47 | 0.78 | 0.31 |
| *Iridomyrmex sp. AA* | 7 |  | 1.62 | 1.11 | 1.67 | 0.63 | 0.66 | 0.86 | 0.20 |
| *Iridomyrmex sp. B* | 66 | 205 | 1.08 | 0.86 | 1.08 | 0.51 | 0.43 | 0.79 | 0.36 |
| *Iridomyrmex sp. C* | 1 | 11 | 1.01 | 0.80 | 0.98 | 0.46 | 0.42 | 0.71 | 0.29 |
| *Melophorus sp. B* |  | 1 | 1.50 | 0.97 | 1.46 | 0.70 | 0.60 | 0.79 | 0.19 |
| *Meranoplus sp. A* | 2 | 5 | 0.76 | 0.79 | 0.69 | 0.80 | 0.74 | 0.80 | 0.05 |
| *Meranoplus sp. B* |  | 2 | 0.85 | 0.92 | 0.84 | 1.04 | 0.97 | 1.01 | 0.04 |
| *Monomorium sp. A* | 34 | 56 | 0.52 | 0.50 | 0.42 | 0.25 | 0.33 | 0.42 | 0.09 |
| *Monomorium sp. B* |  | 1 | 0.41 | 0.40 | 0.28 | 0.21 | 0.27 | 0.32 | 0.06 |
| *Myrmecia sp. A* | 1 |  | 6.82 | 3.69 | 6.90 | 2.51 | 2.75 | 3.82 | 1.07 |
| *Notoncus sp. A* | 4 |  | 1.53 | 1.20 | 1.40 | 0.82 | 0.84 | 1.14 | 0.30 |
| *Ochetellus sp. A* | 72 | 25 | 0.70 | 0.59 | 0.51 | 0.35 | 0.32 | 0.54 | 0.22 |
| *Paratrechina sp. A* | 14 | 9 | 0.82 | 0.64 | 0.82 | 0.41 | 0.43 | 0.51 | 0.08 |
| *Pheidole sp. A* | 8 |  | 0.49 | 0.45 | 0.43 | 0.26 | 0.38 | 0.41 | 0.03 |
| *Pheidole sp. B* |  | 10 | 0.74 | 0.62 | 0.65 | 0.38 | 0.53 | 0.57 | 0.04 |
| *Pheidole sp. C* | 1 | 1 | 0.79 | 0.63 | 0.72 | 0.37 | 0.54 | 0.59 | 0.05 |
| *Prolasius sp. A* | 1 |  | 1.19 | 0.81 | 1.16 | 0.50 |  | 0.64 |  |
| *Prolasius sp. B* | 2 |  | 1.23 | 0.82 | 1.14 | 0.56 | 0.49 | 0.72 | 0.23 |
| *Rhytidoponera metallica gp sp. A* | 9 | 13 | 1.91 | 1.43 | 1.67 | 1.00 | 1.06 | 1.33 | 0.28 |
| *Rhytidoponera metallica gp sp. B* | 30 | 4 | 1.38 | 1.09 | 1.11 | 0.74 | 0.79 | 0.95 | 0.16 |
| *Tapinoma sp. A* | 28 | 41 | 0.61 | 0.54 | 0.52 | 0.29 | 0.27 | 0.44 | 0.17 |
| **Sweden** |  |  |  |  |  |  |  |  |  |
| *Camponotus herculeanus* | 3 | 3 | 3.06 | 2.24 | 2.52 | 1.52 | 1.51 | 2.20 | 0.69 |
| *Formica aquilonia* | 91 | 116 | 2.20 | 1.69 | 2.07 | 1.13 | 1.08 | 1.51 | 0.43 |
| *Formica lemani* | 36 | 71 | 2.01 | 1.42 | 1.81 | 0.91 | 0.83 | 1.20 | 0.37 |
| *Formica lugubris* | 48 | 61 | 2.67 | 2.03 | 2.50 | 1.31 | 1.38 | 1.87 | 0.49 |
| *Leptothorax acervorum* |  | 4 | 1.17 | 0.88 | 0.76 | 0.44 | 0.68 | 0.75 | 0.07 |
| *Myrmica lobicornis* | 1 | 2 | 1.49 | 1.09 | 1.15 | 0.70 | 0.89 | 0.98 | 0.09 |
| *Myrmica ruginodis* | 90 | 5 | 1.77 | 1.27 | 1.44 | 0.76 | 0.93 | 1.05 | 0.12 |
| *Myrmica sulcinodis* |  | 21 | 1.65 | 1.25 | 1.39 | 0.76 | 1.01 | 1.14 | 0.13 |
